# The multidimensionality of sleep in population‐based samples: a narrative review

**DOI:** 10.1111/jsr.13608

**Published:** 2022-04-15

**Authors:** Sterre C. N. van de Langenberg, Desana Kocevska, Annemarie I. Luik

**Affiliations:** ^1^ Department of Epidemiology Erasmus MC University Medical Center Rotterdam the Netherlands; ^2^ Department of Sleep and Cognition Netherlands Institute for Neuroscience Amsterdam the Netherlands; ^3^ Department of Child and Adolescent Psychiatry/Psychology Erasmus MC University Medical Center Rotterdam the Netherlands

**Keywords:** actigraphy, ethnicity, napping, polysomnography

## Abstract

The identification of optimal sleep duration recommendations for the general population has long been an important goal on the public health agenda, as both short and long sleep duration have been linked to unfavourable health outcomes. Yet, sleep is more than duration alone and can be described across multiple domains, such as timing, regularity, satisfaction, alertness, and efficiency. We reviewed observational population‐based studies that examined differences in age, sex, and origin across multiple dimensions of sleep. Reviewed literature suggests an increasing prevalence of insomnia symptoms, shorter and less deep sleep in old age. Overall, women report poorer sleep quality than men despite objective measures revealing shorter and more fragmented sleep in men. Minorities generally have poorer quantity and quality of sleep, but multi‐ethnic studies have reported mixed results regarding the subjective experience of sleep. In sum, effects of age, sex and origin differ across sleep dimensions, thereby suggesting that the multidimensionality of sleep and how these different aspects interact should be studied across individuals. Studies should include both self‐reported measures and objective assessments in diverse population‐based samples, as both aspects are important to understand sleep health in the general population. Data‐driven descriptions could provide researchers and clinicians with insights into how well individuals are sleeping and offer concrete targets for promotion of sleep health across the population.

## 1 INTRODUCTION

Most of us know we sleep roughly one‐third of our lives and that both short and long sleep could pose a risk for our general health and brain function (Grandner, 2017; Hudson et al., 2020). Yet, sleep is a multidimensional construct, which, besides duration, includes aspects such as timing, regularity, satisfaction, and efficiency (Buysse, 2014). Indeed, it is not uncommon that persons report poor sleep quality despite having obtained the recommended number of hours of sleep per night. Ideally, sleep is therefore approached from a framework that considers the extent to which an individual experiences good sleep across multiple domains.

Sleep is a complex interplay of biological and environmental factors spanning a continuum between health and disease (Grandner, 2017). It is also subject to change across the lifespan. The presence of sleep problems, for example, tends to synchronise with major life events (e.g., start of school, pregnancy, retirement), mental and physical illness, lifestyle choices, and sociodemographic variables (Mutambudzi & Van Solinge, 2021; Williamson et al., 2021). Clinicians and expert panels that develop guidelines to promote better sleep at a population level, likely benefit from factoring in these individual, social, and environmental contexts.

Valid data‐driven descriptions of multiple aspects of sleep in the general population can address this continuous nature (Buysse, 2014) and how sleep differs within and between person characteristics, such as age, sex, and origin. A recent meta‐analysis aimed to fill some of these gaps by providing age‐ and sex‐specific reference ranges for sleep duration and sleep complaints based on individual participant data from 1.1 million people from the Netherlands, UK, and United States (Kocevska et al., 2021). In this narrative review, we summarise the literature on the effects of age, sex, and origin on the full range of sleep characteristics in diverse population‐based samples to provide prospects for screening, prevention, and treatment efforts.

## 2 AGE AND SLEEP

### 2.1 Self‐perceived sleep

During the first 2 years of life, sleep is characterised by a steep decline in parent‐observed sleep duration across the 24‐h period, with increasingly more consolidated night‐time sleep and less daytime napping (Galland et al., 2012; Iglowstein et al., 2003; Kocevska et al., 2021). Any remaining daytime sleep in toddlers usually consolidates into one nap during the day, although the exact timing is dependent on cultural factors (Crosby et al., 2005). The prevalence of a variety of parent‐reported sleep problems in young age (0–6 years), including frequent nocturnal awakenings and bedtime resistance, ranges from 10%–20% in the United States, Germany, and Vietnam, and up to 70% in Taiwan and China (Mindell et al., 2010, 2013; Schlarb et al., 2015). These rates generally drop by the time children achieve school age (Galland et al., 2012; Hysing et al., 2016; Uebergang et al., 2017). However, the prevalence of self‐reported insomnia symptoms increases with age from school age onwards: up to 30% of teenagers have insomnia complaints (Calhoun et al., 2014; Kocevska et al., 2021; Schlarb et al., 2015). School‐aged children (5–9 years) and young adolescents also (10–13 years) get increasingly less night‐time sleep on schooldays (Blair et al., 2012; Laberge et al., 2001; Schlarb et al., 2015; Wang et al., 2016), but seem to compensate with extra sleep time (15–20 min) during the weekend (Biggs et al., 2013; Price et al., 2014). The age‐related trend of less adequate and regular sleep peaks in older adolescents (14–19 years), when the gap between weekday and weekend sleep can span up to 1.5 h (Adam et al., 2007; Wolfson & Carskadon, 1998) and the obtained sleep is typically less than the recommended 9 h (Eaton et al., 2010; Keyes et al., 2015; Leger et al., 2012; Maslowsky & Ozer, 2014; Moore et al., 2011; Roberts et al., 2011). The prevalence of daytime tiredness and disturbed mood due to suboptimal sleep is also two‐ to 10‐times higher in late adolescence compared to other age groups (Giannotti et al., 2002; Huang et al., 2010; Kronholm et al., 2015; Lewien et al., 2021).

In adults, self‐reported sleep duration shortens with age, while current recommendations for optimal sleep duration remain largely unaltered from the age of 18 years onwards (Hirshkowitz et al., 2015; Hublin et al., 2020; Kocevska et al., 2021; Suh et al., 2020; Tonetti et al., 2008). The prevalence of self‐reported short sleepers (<6 h) has been estimated to increase from 6.8% in young (18–34 years) and middle‐aged adults (35–54 years) up to 25% in elderly persons (>55 years) in the European population and up to 30% in the United States and Japanese elderly population (Kocevska et al., 2021; Kronholm et al., 2006). Of note, several studies also find that the percentage of long sleepers among old adults (>60 years) is about twice as high compared to younger age groups (Cheng et al., 2017; Kronholm et al., 2006; Krueger & Friedman, 2009; Nakakubo et al., 2022). These findings thereby suggest more variation in sleep duration and time in bed at old age. Self‐reported sleep efficiency shows a stable age‐related trend throughout most of adult life but declines with age in elderly adults (Kocevska et al., 2021). Similarly, sleep quality remains stable within young and middle‐aged adults (Kocevska et al., 2021). The prevalence of poor sleep quality, sleep problems and hypnotic medication use is significantly higher in older adults (>65 years; Grandner, Martin, et al., 2012; Nakakubo et al., 2022; Phillips & Mannino, 2005; Rediehs et al., 1990; Soldatos et al., 2005; Suh et al., 2020; Unruh et al., 2008). Specifically, elderly persons experience more difficulty maintaining sleep (20%–39% versus 9%–16%) and early morning awakenings (19%–24% versus 10%–21%) than younger and middle‐aged persons (Foley et al., 1995; Kocevska et al., 2021). In contrast, rates of complaints related to non‐restorative sleep, daytime tiredness or impaired daytime functioning are generally lower in old adults (Grandner, Martin, et al., 2012; Kaplan et al., 2017; Liu et al., 2000; Phillips & Mannino, 2005; Soldatos et al., 2005; Unruh et al., 2008). Notably, older individuals show better sleep across various domains when measures are adjusted for general health status (Phillips & Mannino, 2005).

### 2.2 Objective assessments of sleep

Objective estimations of sleep duration and efficiency are generally lower than indications by subjective measures, but both reveal patterns of shorter and more fragmented sleep with increasing age across the lifespan (Evans et al., 2021; Kocevska et al., 2021; Ohayon et al., 2004). At 6 months of age, the average daily sleep duration in infants has been estimated at 11 h by actigraphy, of which ~25% is obtained through multiple daytime naps (Tsai et al., 2022). In the first 3 years of life, daytime sleep duration rapidly decreases and is replaced for longer, more consolidated night‐time sleep (Galland et al., 2012). However, objective assessments of sleep characteristics, particularly for sleep onset latency, are generally lacking for infants and toddlers. In children aged between 4 and 12 years, an age‐related decline in the total duration of sleep has consistently been observed (Evans et al., 2021; Pesonen et al., 2014; Vaipuna et al., 2018). This decline continues in teenagers, but measures become increasingly variable within and between individuals with the onset of puberty (Kocevska et al., 2021; Moore et al., 2011). In adults, objective estimates of sleep duration and sleep efficiency slightly decrease with age (Evans et al., 2021; Floyd et al., 2000; Kocevska et al., 2021; Moraes et al., 2014; Nunez et al., 2021; Redline et al., 2004). Additionally, sleep in ageing individuals is typically characterised by a slight increase in onset latency (10–15 min increase throughout adult life), moderately more frequent awakenings, substantially longer wake time during the night (mean increase of 7–10 min with 10 years of age), and an increasingly higher arousal index (mean increase of 2.5 arousals/h with 10 years of age) (Bonnet & Arand, 2007; Floyd et al., 2000; Moraes et al., 2014; Unruh et al., 2008). The prevalence of daytime‐only napping (27%) and daytime or evening napping (58%) is especially high among elderly adults (Dautovich et al., 2008).

When assessing children's sleep with polysomnography (PSG), the first cycle of non‐rapid eye movement (NREM) sleep during the night is particularly rich in slow‐wave sleep (SWS) with high delta electroencephalography (EEG) power (Bonnet & Arand, 2007; Montgomery‐Downs et al., 2006). As children progress into adolescence, the percentage of SWS decreases, such that lighter sleep stages (N1 and N2) gradually take up more of the NREM sleep (Baker et al., 2016; Montgomery‐Downs et al., 2006; Ricci, He, Fang, et al., 2021). At the age of 18 years, the total duration of SWS is reduced by almost half (Purcell et al., 2017). The macrostructure of sleep continues to change according to an age‐specific pattern of increasingly lighter sleep, i.e., less SWS (Moore et al., 2011; Moraes et al., 2014; Ohayon et al., 2004; Redline et al., 2004; Ricci, He, Fang, et al., 2021; Yoon et al., 2021). Sleep spindle frequency increases and EEG power decreases in the transition from childhood to adolescence (Purcell et al., 2017), whereas both the frequency and power of spindles decline in adults. REM sleep initially becomes longer but more fragmented with age during childhood and adolescence, remains stable in young and middle‐aged populations, and then slightly shortens in people aged ≥60 years (Moraes et al., 2014; Ohayon et al., 2004; Pesonen et al., 2019; Redline et al., 2004; Yoon et al., 2021).

## 3 SEX AND SLEEP

### 3.1 Self‐perceived sleep

Population‐based evidence for sex differences in self‐perceived sleep has been accumulating over recent decades. Overall, studies suggest that the prevalence of perceived poor sleep is 1.2–2‐times higher among adolescent and adult women (Doi et al., 2001; Kocevska et al., 2021). Sex differences in subjective sleep have not been reported consistently for infants and children (Biggs et al., 2013; Galland et al., 2012; Iglowstein et al., 2003; Russo et al., 2007; Schlarb et al., 2015), but parents' reports on sleep problems indicate that certain issues, such as frequent night‐time awakenings, bed‐time resistance, and daytime tiredness, are experienced disproportionally more often by preschool boys (Kocevska et al., 2021; Lewien et al., 2021; Li et al., 2014; Uebergang et al., 2017). From the onset of puberty onward, women typically report more insomnia symptoms than men (Adams et al., 2017; Asghari et al., 2012; Dollman et al., 2007; Giannotti et al., 2002; Huang et al., 2010; Iglowstein et al., 2003; Klackenberg, 1982; Kronholm et al., 2015; Laberge et al., 2001; Lee et al., 1999; Luntamo et al., 2012; Ohayon et al., 2000; Pallesen et al., 2008; Schlarb et al., 2015; Tang et al., 2017; Tonetti et al., 2008). Middle‐aged and elderly women show a heightened prevalence of experiencing difficulties with initiating sleep (female: 13%–29% versus male: 5%–18%), maintaining sleep (female: 10%–31% versus male: 8%–20%) and early‐morning awakenings (female: 11%–28% versus male: 9.5%–18%) (Groeger et al., 2004; Kocevska et al., 2021; Middelkoop et al., 1996).

Following a night of poor sleep, women appear to be at elevated risk of experiencing impairments throughout the day, e.g., (excessive) daytime tiredness, apathetic or irritable mood and reduced vigilance during daily activities, compared with men (Doi et al., 2001; Grandner, Martin, et al., 2012; Huang et al., 2010; Kronholm et al., 2015; Suh et al., 2020). Furthermore, studies have reported a higher prevalence of non‐restorative sleep and self‐perceived inadequate sleepers in women compared to men (Eaton et al., 2010; Grandner, Jackson, et al., 2015; Ohayon et al., 2000; Peltzer, 2017; Tang et al., 2017; Um & Um, 2015). Notably, adult women report the use of sleep medication more frequently compared to men (Fernandez‐Mendoza et al., 2012; Kocevska et al., 2021; Middelkoop et al., 1996). Yet, the ranges of self‐reported sleep duration reported in the literature indicate that adolescent girls (7.2–8.4 h) and boys (7.4–8.5 h) largely get similar amounts of sleep per night (Adam et al., 2007; Kocevska et al., 2021; Matthews et al., 2014; Spilsbury et al., 2004), and that adult women generally sleep slightly longer per night (6.7–7.4 h) than men of similar age (6.8–7.25 h) (Gilmour et al., 2013; Kocevska et al., 2021; Liu et al., 2021; Singh et al., 2020; Tang et al., 2017).

### 3.2 Objective assessments of sleep

Common sleep parameters display sex differences that remain significant upon adjustment for sociodemographic and socioeconomic factors when measured with PSG and actigraphy. Objectively estimated night‐time sleep duration shows that women might sleep up to 45 min longer than men (James et al., 2020; Lauderdale et al., 2006; Matthews et al., 2014; Moore et al., 2011; Roehrs et al., 2006; Wendt et al., 2020). Sleep onset latency is estimated to be 5 min longer for adult women compared with men, which objectively confirms the higher ratings of “difficulties with falling asleep” in women from puberty onwards (Lauderdale et al., 2006; Roehrs et al., 2006). In contrast to the higher prevalence of insomnia symptoms among women, efficiency and continuity of sleep as measured by actigraphy and PSG indicate less fragmented sleep in women compared to men (Baker et al., 2016; Kocevska et al., 2021; Lauderdale et al., 2006; McCrae et al., 2008; Roehrs et al., 2006; Wendt et al., 2020).

In‐depth research on sleep architecture provides additional support that women's sleep is more consolidated and resilient to external stress factors (Bixler et al., 2009). Specifically, PSG studies reveal that adult women have lower percentage Stages 1 and 2 of NREM sleep at the likely benefit of an increased length and intensity of their SWS, at least until they reach menopause (Choi et al., 2019; Roehrs et al., 2006; Yoon et al., 2021). Although the percentage of SWS and the slow‐wave activity power markedly decrease in both men and women as they get older, the sleep of men is characterised by a steeper decline in these measures and a higher arousal index (Baker et al., 2016; Mallampalli & Carter, 2014; Moraes et al., 2014; Redline et al., 2004; Ricci, He, Fang, et al., 2021).

In terms of spindle characteristics, women's fast sleep spindles typically display greater density and enhanced oscillations compared to men's (Choi et al., 2019; Merikanto et al., 2017; Ricci, He, Calhoun, et al., 2021). Spectral analyses show that absolute rather than relative spectral power varies by sex, with women showing greater power densities across all frequency bands, except for Beta power, throughout the night (Yoon et al., 2021). Relative power in Alpha, Beta and Sigma ranges is higher in adult women, which has been linked to insomnia symptoms and may better account for sex‐specific anatomical variations (e.g., head size, skull, or skin thickness) (Choi et al., 2019). However, it is still debatable whether the observed differences in neural activity mirror differential regulatory sleep processes or confounding of EEG measurements (Roehrs et al., 2006).

## 4 ORIGIN AND SLEEP

### 4.1 Self‐perceived sleep

Overall, population‐based studies show that minorities are less likely to get healthy sleep across various sleep dimensions. Research into disparities in sleep duration has primarily come from North America and focusses on origin. The prevalence of short and long sleepers is higher in Blacks/African Americans, Hispanic/Latinos, Asians, Native Hawaiian/Pacific Islanders, and American Indians/Alaskan Natives, compared with Whites, Americans of mixed origin and foreign‐born US residents (Carnethon et al., 2016; Cunningham et al., 2016; Fox et al., 2018; Gamaldo et al., 2015; Hale & Do, 2007; Liang et al., 2020; Matthews et al., 2018; Nunes et al., 2008; Singh et al., 2020; Whinnery et al., 2014). European studies have additionally reported that the prevalence of not getting the recommended hours of sleep in ethnic minorities might be up to two‐times higher than in ethnic‐Europeans (Anujuo et al., 2014; Malone et al., 2016). In South Africa, the White population is considered a minority and is more likely to report short sleep duration (16.9%–18.8%) compared with Black people (6.5%–7%), Asian people (14.5%–17.1%) and people of mixed origin (12.7%–15.6%) (Peltzer, 2017). In the highly multi‐ethnic and multicultural population of Singapore, people of Malay and Indian origin are considered minorities and report significantly less nocturnal sleep and 20 min longer daytime nap duration than Chinese people (Cheng et al., 2017). Within these studies, the gap in self‐reported sleep duration between minority and majority groups can stretch from 24 min up to >1 h.

Notwithstanding the consistent finding of less sleep among these minorities, the role of origin in the experience of sleep is complex. On the one hand, population‐based studies report that Black and Asian people experience poorer overall sleep quality than Whites (Chen et al., 2015; Grandner et al., 2013; Patel et al., 2010). Black people are more likely and Asian people less likely to experience daytime sleepiness, compared to Whites (Baron et al., 2010; Chen et al., 2015). On the other hand, several studies find a higher prevalence of insomnia complaints and non‐restorative sleep among White individuals or those with a mixed background (Grandner et al., 2010; Matthews et al., 2018; Phillips & Mannino, 2005; Singh et al., 2020). In general, epidemiological studies on the link between origin and the nature and prevalence of sleep complaints yield rather mixed results (Grandner et al., 2010; Halder et al., 2015; Matthews et al., 2018; Mezick et al., 2008). Geographical location and neighbourhood characteristics might also affect sleep (Gamaldo et al., 2015; LaVeist et al., 2011). Large population‐based studies show substantial differences in how individuals sleep around the world (Gradisar et al., 2011; Soldatos et al., 2005). In Asia, preschool children (0–6 years) generally go to bed later, sleep fewer hours per night, have more parent‐reported sleep problems, show higher rates of daytime tiredness, and preserve the habit of napping for longer compared with their peers from North America, Europe, and Oceania (Galland et al., 2012; Mindell et al., 2010, 2013). Research shows similar patterns for schoolchildren and adolescents (Gradisar et al., 2011; Liu et al., 2005). Adults from East and Southeast Asia (Dong et al., 2018; Ryu et al., 2011; Tang et al., 2017; Thichumpa et al., 2018; Um & Um, 2015; Wang et al., 2020; Wu et al., 2018), Central and Southern America (Carrillo‐Larco et al., 2014; Lima et al., 2012; Neutzling et al., 2020; Soldatos et al., 2005), Sub‐Saharan Africa (Ade et al., 2021; Peltzer, 2017; Soldatos et al., 2005), The Middle East (Asghari et al., 2012; Chami et al., 2020), and urban regions in North America (Grandner, Smith, et al., 2015) are more likely to report sleep of poor quality (range 28%–50%, generally higher in rural areas) and short duration (range 12%–39%, generally higher in metropolitan urban areas) than adults residing in Europe, Japan, Australia and non‐urban regions in North America (Adams et al., 2017; Doi et al., 2001; Kocevska et al., 2021; Lakerveld et al., 2016; Liu et al., 2000). In most Western countries, the average self‐reported sleep duration (from ~7 h/night in Japan to 8.5 h/night in Portugal) and indicators of poor sleep quality (range 9%–30%, depending on the indicator assessed) are relatively comparable between populations (Adams et al., 2017; Doi et al., 2000; Groeger et al., 2004; Kocevska et al., 2021; Kronholm et al., 2006; Soldatos et al., 2005). In most European countries, China and South Africa, the prevalence of complaints related to frequent nocturnal awakenings and prolonged sleep onset latency is highest, whereas adults in Brazil and Japan most frequently report insufficient sleep duration (Soldatos et al., 2005). Furthermore, rates of both inadequate sleep and insomnia symptoms are 1.2–3‐times higher in the United States compared to European samples (Kocevska et al., 2021).

### 4.2 Objective assessments of sleep

Physiological measures of sleep further support that minorities have poorer values on multiple dimensions of sleep. Population‐based evidence almost exclusively comes from analyses conducted in samples from the United States (100–11,000 individuals), but consistently shows that African Americans have longer sleep onset latency and wake after sleep onset, lower sleep efficiency, and up to 40 min shorter total sleep duration than non‐minority individuals (Matthews et al., 2014; Mezick et al., 2008; Moore et al., 2011; Rao et al., 2009; Redline et al., 2004; Wendt et al., 2020). Similar results have been observed for American Indians and Asians (Redline et al., 2004).

In the American population, assessments of sleep macrostructure by PSG have further underpinned that minorities generally spend more time in lighter sleep stages (N1 and N2) and less time in SWS compared with Whites and Hispanics (Lauderdale et al., 2006; Mezick et al., 2008; Purcell et al., 2017; Rao et al., 2009; Redline et al., 2004; Yoon et al., 2021). At a young age, the SWS of African Americans is characterised by lower estimates of spindle density, amplitudes, and duration, but does not substantially differ in terms of spindle frequency compared with Whites (Purcell et al., 2017). These differences exist with and without controlling for age, sex, and socioeconomic status. Additionally, NREM sleep of African Americans displays lower absolute and relative EEG power across the delta to sigma frequency range (Lauderdale et al., 2006; Purcell et al., 2017). However, a higher arousal index is observed in Whites, perhaps indicating a lower threshold to interruption by the external environment (Baker et al., 2016; Redline et al., 2004). Research to date has not shown variations in percentage REM sleep by origin (Mezick et al., 2008; Redline et al., 2004).

## 5 DISCUSSION

Within this review, we showed that characteristics of sleep show considerable variation across sex, age, and origin in population‐based samples. A one‐size‐fits‐all approach is therefore unlikely to help prevention and treatment efforts. Moreover, we showed that the variation in sleep characteristics is not uniform across demographic groups. For instance, elderly persons tend to show shorter sleep but do yet not have poorer sleep quality across all domains. Population‐based research should therefore move away from assessing sleep duration or self‐reported sleep quality only, and instead approach sleep as a multidimensional construct, although the interaction between sleep characteristics warrants further attention.

To date, epidemiological evidence has rather consistently shown that with older age, sleep duration shortens, the prevalence of self‐reported insomnia symptoms increases, and objectively assessed sleep becomes lighter and more fragmented. This worse quantity and quality of sleep may arise from a variety of causes, including comorbidity, retirement, medication side‐effects, change in circadian rhythms, and changes in social patterns (Foley et al., 1995; Maggi et al., 1998). In contrast, daytime consequences of inadequate sleep, including excessive daytime tiredness, reduced vigilance, and impaired mood and cognitive functioning, are less likely to be reported by older individuals in population‐based samples. Potentially, young and middle‐aged adults have higher rates of reporting daytime effects because of more demanding schedules and societal obligations, such as child‐rearing and work, compared with other age groups (Grandner, Jackson, et al., 2015).

The reviewed literature indicates that sex differences already emerge with the onset of puberty, but it seems that women are particularly prone to report difficulties with initiating and maintaining sleep and experience more problems with waking up too early from middle‐age onwards. Sex‐specific variation in sleep behaviour (i.e., actigraphy) or physiology (i.e., PSG) are not likely to explain the poorer self‐perceived sleep in women, because objective measures point to longer, deeper, and less fragmented sleep in women. The elevated incidence and persistence of insomnia symptoms in women could result from increased vulnerability of women's sleep to stressful life events or heightened social pressure (Suh et al., 2018). Alternatively, it could also be related to higher levels of systemic inflammation (Dolsen et al., 2019) and differential exposure to hormones (e.g., women have higher levels of cortisol upon awakening) (Mong & Cusmano, 2016; Suh et al., 2018). Of note, the few studies that directly examined potential determinants of sex differences in sleep, have reported conflicting findings and primarily focussed on self‐reported sleep (Pengo et al., 2018).

This review also indicates that minorities and socioeconomically disadvantaged populations generally have higher prevalence of short and long sleep duration, prolonged sleep onset latency, less consolidated sleep, worse self‐perceived sleep quality, and lower sleep efficiency. Whites tend to report more complaints with respect to non‐restorative or insufficient sleep. These differences might in part be explained by the design of our sleep measures, including questionnaires, actigraphy and PSG, which have typically been designed and validated within Western and White populations. However, these differences might also be explained by differences in genetics, stress response, and general health status (Grandner, Jackson, et al., 2015; Halder et al., 2015; Malone et al., 2016; Rao et al., 2009). Additionally, societal integration pressure or discrimination could underlie the observed variation in sleep by minority status (Alcantara et al., 2019; Grandner, Hale, et al., 2012; Hill et al., 2021; Pandi‐Perumal et al., 2017; Tomfohr et al., 2012; Van Dyke et al., 2016). Health status and stress levels are also closely related to socioeconomic risk factors, such as low income, low education level, and poor living conditions, which are likely to be more prevalent among minorities (Billings et al., 2020; Grandner et al., 2013; LaVeist et al., 2011; Mezick et al., 2008; Pandi‐Perumal et al., 2017).

Based on this review, we would like to raise three important practice points for research (Table 1). First, we emphasise the need for assessment of the multidimensionality of sleep (including regularity, satisfaction, alertness, duration, efficiency) (Buysse, 2014). Although large population‐based studies of the previous century have had the tendency to reduce the study of sleep to the study of sleep duration alone, we increasingly see studies that assess multiple aspects of sleep in large population‐based samples. This improves our insight in the associations of sleep with health and disease and offers new avenues for prevention efforts. However, sleep has is yet to be studied as a true multidimensional construct where one considers how the distinct aspects of sleep interact. A small body of prior research suggests that even when associations with single sleep components or sleep dysfunction are absent, composite sleep health measures may be predictive of determinants and consequences (Brindle et al., 2018; Lee et al., 2022). Health outcomes of individuals with different multidimensional profiles of sleep health are also likely to differ (e.g., a short sleeper with a regular schedule that is satisfied with their sleep likely carries different risks than a person experiencing insomnia problems with sufficient sleep duration). A clear definition of sleep health is thus urgently needed.

**TABLE 1:** Practice points and future directions

Sleep should be assessed with complementary methods combining self‐report with objective assessments of sleep (e.g., actigraphy, polysomnography) and to be able to assess all relevant domains. The multidimensionality of sleep should be considered in all types of research, including population‐based samples as this can help us define treatment and prevention targets. Future studies should be conducted in more diverse samples (e.g., with respect to age, sex and gender, race/ ethnicity, and geographical location) to enhance our understanding of sleep health and its effects across various groups.

Second, to ensure thorough assessment of the multidimensionality of sleep, a combination of multiple measurement methods should be used as each may cover an important aspect or level of the multidimensional construct of sleep. Self‐report measures give a valid insight in how individuals perceive their sleep, which may be most important to well‐being. However, self‐report measures are prone to recall bias and the interpretation of sleep questionnaire surveys depends on how and by whom these are designed (Fabbri et al., 2021). This might particularly harm findings on the effects of sleep duration, which have typically been assessed by a single self‐reported question in population‐based studies (e.g., how many hours of sleep do you usually get per night?). However, this simple question is unlikely to reflect actual sleep duration. Anecdotally, most sleep researchers will have encountered self‐reported nocturnal sleep durations surpassing the time spent in bed. This could have multiple causes, for example because persons might not be aware of being awake at night or because of night‐to‐night variability. Research has also suggested that the correlation between self‐reported and actigraphy‐estimated sleep is only moderate and systematically biased (Lauderdale et al., 2008). Objective measures can complement to self‐reported sleep. PSG is still the “gold‐standard” method in sleep research, but is costly, time‐consuming, and burdensome for the participant, thereby making it more difficult to apply in large population‐based samples (Marino et al., 2013). Use of ambulant PSG can partially overcome these problems. Actigraphy is advantageous in terms of costs, time, and applicability, and has already been used in many large population‐based samples. It additionally allows for measuring sleep for longer periods of time. Yet, it cannot fully replace PSG (Marino et al., 2013). These measures should therefore ideally be combined to estimate multilevel, multidimensional sleep health.

Third, we emphasise the need for study of diverse, inclusive population samples. Although population‐based samples have been suitable for the examination of sleep across age and sex, this cannot be said with respect to origin. Sleep is increasingly studied among minorities and in different geographical regions, but reports are still limited compared to the data available from Western countries and in Whites. Moreover, up until today many of our measurements have been developed and validated in Western and White participants, which may not adequately reflect sleep patterns in other populations. Future research across all origins with valid tools is therefore urgently needed.

In conclusion, multilevel multidimensional population‐based approaches could help to provide a framework to conceptualise how sleep health varies at the individual, social, and societal level. By combining and refining multiple sleep measurement tools for population‐based studies, researchers will get closer to comprehensively assessing multiple sleep characteristics among large, diverse populations across the world. In turn, these data‐driven descriptions of multidimensional sleep in the population overall could provide researchers, clinicians, and policy makers with concrete targets for promotion of sleep health and prevention of disease.

## CONFLICT OF INTEREST

The authors declare no conflict of interest.

## AUTHOR CONTRIBUTIONS

Sterre C. N. van de Langenberg reviewed the literature. Sterre C. N. van de Langenberg, Desana Kocevska, and Annemarie I. Luik drafted and critically revised the manuscript.

## Data Availability

Data sharing not applicable to this review article as no datasets were generated or analysed during the current study.
